# Two-stage strategies to detect gene × gene interactions in case-control data

**DOI:** 10.1186/1753-6561-1-s1-s135

**Published:** 2007-12-18

**Authors:** Amina Barhdadi, Marie-Pierre Dubé

**Affiliations:** 1 Department of Medicine, Université de Montréal and the Research Centre of the Montreal Heart Institute, 5000 Bélanger, Montréal, QC H3W1R3, Canada

## Abstract

Large genetic association studies based on hundreds of thousands of single-nucleotide polymorphisms (SNPs) are a popular option for the study of complex diseases. The evaluation of gene × gene interactions in such studies is a sensible method of capturing important genetic effects. The number of tests required to consider all pairs of SNPs, however, can lead to a computational burden, and efficient strategies to reduce the number of tests performed are desirable. In this study, we compare two-stage strategies for pairwise SNP interactions testing. Those approaches rely on the selection of SNPs based on the single-locus test results obtained at the first stage. In the *simultaneous *approach, SNPs that fall below the marginal significance thresholds (*p *= 0.05 and *p *= 0.1) in stage 1 are selected and tested for within-group pairwise interaction in stage 2. With the *conditional *approach, SNPs that reach Bonferroni-adjusted significance at the first stage are tested in pairwise combinations with all SNPs in the data set. We compared the performance of those strategies by using Replicate 1 of the simulated data set of the Genetic Analysis Workshop 15 Problem 3. Most interactions detected resulted from SNP pairs within 1000 kb of each other. The remaining were false positives involving SNPs with excessively strong marginal signals. Our results highlight the need to account for locus proximity in the evaluation of interaction effects and emphasize the importance of marginal signal strength in logistic regression-based interaction modeling. We found that modeling additive genetic effects alone was sufficient to capture underlying dominance interaction effects in the data.

## Background

Genetic association is an increasingly popular method to identify genetic determinants of common diseases. Traditional single-locus association tests evaluate the marginal effects of each marker. It is to be expected, however, that the genetic susceptibility of complex traits would result from the interplay of several factors, including gene × gene interactions. As such, analytical approaches that consider single-nucleotide polymorphism (SNP) interaction effects have the potential to provide more power, especially when susceptibility genes have small or undetectable marginal effects.

The number of possible SNP pairs grows rapidly with respect to the number (*n*) of SNPs in a study, following *n*(*n *- 1)/2, and testing for the entire probability space for SNP × SNP interaction can become computationally unfeasible or cumbersome. Some promising two-stage approaches have been proposed [1,2] to alleviate the problem, and we were particularly interested in comparing the performance of three of those by using replicate 1 of the simulated data set from Problem 3 of the Genetic Analysis Workshop 15 (GAW15).

The two-stage strategies that we use rely on the selection of SNPs based on their marginal single-locus test results obtained in the first stage. The *simultaneous *design is an approach that will test for interaction effects only between SNPs with *p*-values that fall below a pre-determined marginal significance threshold. We here compare the performance of the simultaneous design with thresholds of *p *= 0.05 and *p *= 0.1. It is expected that the more permissive threshold would offer greater detection power for an underlying model with weaker marginal signals.

We also evaluated the performance of the *conditional *two-stage design. The conditional approach tests for interaction effects of SNPs that reach global significance (after multiple comparison adjustment) at the first stage, in pairwise combination with all of the SNPs in the data set. This approach, as compared to the simultaneous design, has the advantage of including interactions where one of the SNPs would not show any evidence for a main effect.

Interaction effects have previously been modeled by logistic regression as described by Cordell [3]. We were interested in comparing a model that includes both dominance and additive interaction terms as compared to modeling only the additive effects. In previous work published by North et al. [4], it was suggested that the additive-only model was sufficient to detect most interaction models, whereas the combined additive and dominance effect model had the disadvantage of increasing the degrees of freedom of the logistic model and increasing the number of interaction terms to test for in each model construction, thereby imposing an unnecessary additional multiple comparison burden.

## Methods

To obtain a sample of cases and controls, we randomly chose one case from each simulated affected sib pair from Replicate 1 of GAW15 Problem 3. Our sample consisted of 1500 cases with rheumatoid arthritis and 2000 controls. Available covariates for controls included sex, lifetime smoking, DR alleles and age. We used only sex, smoking, and DR alleles as significant covariates in our adjusted model. We ran our interaction models once without covariates and once with the covariates. We used the genome-wide 10 K simulated SNP chip set with 9187 polymorphic SNPs. There are no missing data and no errors in this data set. Analyses were performed without knowledge of the simulated answers. Computations were made with SAS v.9.1.3 (SAS Institute Inc., Cary, NC, USA) on WinXP and Sun (SAS code available upon request, see ).

### First stage

We tested 9187 SNPs for association by logistic regression modeling according to:

log(*r*/(1 - *r*)) = *μ *+ *ax *+ *dz*,

where *r *is the probability of each individual being a case, *x *and *z *are dummy variables with *x *= 1, *z *= -0.5 for one homozygote genotype, *x *= 0, *z *= 0.5 for the heterozygote genotypes, and *x *= -1, *z *= -0.5 for the other homozygote type. *μ *Corresponds to the mean effect. The terms *a *and *d *correspond to the additive and dominance coefficient estimates of the tested SNP. The *p*-values of the global model were considered. The additive effects model with adjustment for covariates was modeled as:

log(*r*/(1 - *r*)) = *μ *+ *ax *+ *sex *+ *smoking *+ *DRalleles*

following the same notation. For this model, the *p*-value of the additive coefficient *a *was used. Bonferroni correction was used for the conditional design.

### Second stage

We also used logistic regression to model the effect of genotypes and SNP × SNP interactions on the disease risk. We included terms that allow for the estimation of additive effects and dominance effects for each SNP locus, along with the inter-SNP additive and dominance interactions. The full interaction model, following Cordell's notation [3] is:

$$\log(\frac{r}{1-r})=\mu +a_{1}x_{1}+d_{1}z_{1}+a_{2}x_{2}+d_{2}z_{2}+i_{aa}x_{1}x_{2}+i_{ad}x_{1}z_{2}+i_{da}z_{1}x_{2}+i_{dd}z_{1}z_{2},$$

where *r *is the probability of each individual being a case, *x*_*i *_and *z*_*i *_are dummy variables with *x*_*i *_= 1, *z*_*i *_= -0.5 for one homozygote genotype, *x*_*i *_= 0, *z*_*i *_= 0.5 for the heterozygote genotypes, and *x*_*i *_= -1, *z*_*i *_= -0.5 for the other homozygote. *μ *Corresponds to the mean effect; the terms *a*_1_, *d*_1_, *a*_2_, *d*_2_, are the dominance and additive effect coefficients of the two SNPs and *i*_*aa*_, *i*_*ad*_, *i*_*da*_, *i*_*dd*_, represent their interaction coefficients. The additive effects-only interaction test was modeled as:

log(*r*/(1 - *r*)) = *μ *+ *a*_1_*x*_1 _+ *a*_2_*x*_2_*+ i*_*aa*_*x*_1_*x*_2 _+ *sex *+ *smoking *+ *DRalleles*

according to the same notation.

SNPs were selected in the first stage for marginal significance levels up to 0.1 and 0.05 for the simultaneous design and up to the Bonferroni adjusted threshold for the conditional method. In the second stage, the *p*-values of the four interaction terms *i*_*aa*_, *i*_*ad*_, *i*_*da*_, and *i*_*dd *_were considered in the full model [Eq. (3)], and the *p*-value of the interaction coefficient *i*_*aa *_was used in the additive-only model [Eq. (4)]. Bonferroni correction was used by considering the total number of valid interaction term tests. Valid interaction tests refer to those for which the problem of quasi-separation does not occur when using the logistic regression model. We also used an interaction model in which the additive effects are considered without covariates (results not shown), in this case, the *p*-values of the one interaction term (*i*_*aa*_) was used.

## Results

### Stage 1

**Figure 1:**
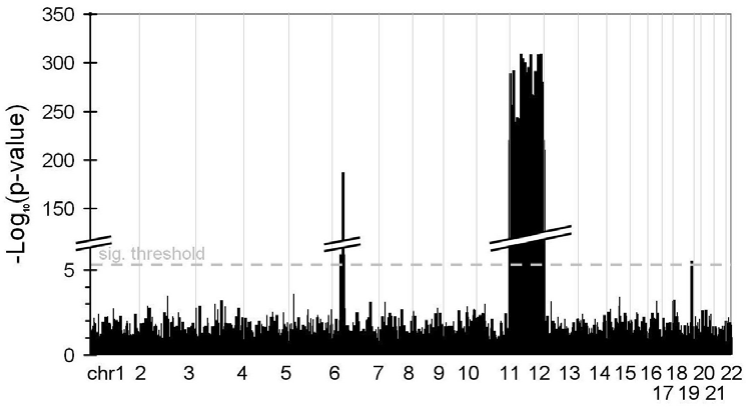
Marginal SNP association results for 1500 cases of rheumatoid arthritis and 2000 controls from the simulated Replicate 1 of GAW15, detected by using an additive and dominance genetic effects logistic regression model in [Eq. (1)].

First, using the logistic model with both dominance and additive effects, without adjustment for covariates [Eq. (1)], we obtained 1361 SNPs that fell below the threshold of *p *= 0.1, and 930 SNPs below the threshold of *p *= 0.05. The tests for all possible pairwise combinations involved (1361 × 1360)/2 = 925,480 interaction tests for the threshold of 0.1 and (930 × 929)/2 = 431,985 tests for the 0.05 threshold. For the conditional design, Bonferroni correction left 443 significant SNPs: 428 of which are located on chromosome 11, 14 SNPs on chromosome 6, and 1 SNP on chromosome 18. These results correspond to the detectable marginal SNP effects simulated in the data set (Fig. 1). The number of pairwise combinations for the conditional design sums up to 443 × 9186 = 4,069,398.

For the logistic model with adjustment for sex, smoking, and DR alleles [Eq. (2)], we considered only the additive effects. 1319 SNPs fell below the threshold of *p *= 0.1, and 894 SNPs below the threshold of *p *= 0.05. Using the global Bonferroni correction, 398 SNPs were found significant, of which, 395 were on chromosome 11 and 3 SNPs were on chromosome 6.

### Interaction test results

When using the full model with dominance and additive genetic terms [Eq. (3)] with the simultaneous design, five significant interactions were found by the dominance interaction term (*i*_*dd*_) (Table 1), but all five were also detected by the coefficient of additive effects (*i*_*aa*_). The same model detected 57 interactions with the *i*_*aa *_term, and the logistic model for additive effects-only (not shown) detected 7 more interactions, due to the reduction in the multiple testing correction. Based on this result, we proceeded to conduct the analysis with adjustment for covariates by using only the additive interaction model [Eq. (4)]. The use of either thresholds at *p *= 0.05 or *p *= 0.1 for the simultaneous design provided exactly the same interaction results for the full model without covariates [Eq. (3)], but a few more were found with the additive model with covariates [Eq. (4)] when using the threshold of *p *= 0.1.

**Table 1:** Number of significant interactions detected in Stage 2 of the simultaneous and the conditional designs by logistic regression modeling [Eq. (3) and (4)].

	Full model without covariates [Eq. (3)]				Additive effects with covariates [Eq. (4)]
		
	*i*_*aa*_	*i*_*ad*_	*i*_*da*_	*i*_*dd*_	*i*_*aa*_
Simultaneous design *p *< 0.05	863,970 tests				399,171 tests
<1,000 kb	46	0	0	1	206
>1,000 kb	11	0	0	0	70
Simultaneous design *p *< 0.10	925,480 tests				869,221 tests
<1,000 kb	46	0	0	1	209
>1,000 kb	11	0	0	0	93
Conditional design	4,069,398 tests				3,656,028 tests
<1,000 kb	49	0	0	0	353
>1,000 kb	75	0	0	4	617

Overall, a large fraction of the interactions detected are between SNPs that are in close physical proximity. A closer look at the results obtained with the full model [Eq. (3)] reveals that 129 different SNP × SNP interactions were found with either the conditional or simultaneous design. After eliminating the 52 interaction results between SNP < 1000 kb, 77 interactions remained, 75 of which include a chromosome 11 SNP around Locus F, 6 of those are proximity interactions on chromosome 11, and 69 are false positive between chromosome 11 and 1. This chromosome 11 region had excessively high marginal signals (*p*-values < 10^-200^), which we believe to be responsible for generating false-positive interaction signals (Fig. 1). The other two interactions were between SNPs in high linkage disequilibrium on chromosome 6, also with excessively high marginal signals. A similar pattern was found for the additive effects model with covariates [Eq. (4)] in which 970 interactions were found with the conditional design, of which 430 were between two SNPs on chromosome 11, and 540 were between one SNP on chromosome 11 and the other on chromosome 1.

## Discussion

The simulated data set contained five genetic loci associated with the case-control status (Loci A to F). According to the simulation schema of Problem 3, a simulated genetic interaction effect between Locus A on chromosome 16 and Locus C on chromosome 6 was modulated by the DR alleles. In Stage 1, we were able to detect the simulated Loci C on chromosome 6, D on chromosome 6, and E on chromosome 18, which passed Bonferroni correction in both logistic regression models with adjustment for covariates and without adjustment for covariates (Fig. 1). Locus F on chromosome 11 was detected by the additive and dominance model. Locus A on chromosome 16 was only identified by the logistic model with covariates in Stage 1 of the simultaneous design with a *p*-value < 10^-3^. Overall, we detected SNP × SNP interactions involving C*C, F*F and F*chromosome 1 loci.

The simultaneous method optimizes the search for interaction effects between SNPs that provide some indication of marginal associations. This situation is most appropriate for disease models with additive polygenic effects. The advantage of the conditional design, on the other hand, is that it can capture interactions with loci that have no marginal effects, and as such is expected to be valid over a wider range of disease models. With our data, neither approach was able to capture the real underlying interaction between Loci A and C. Posterior testing for interaction between these loci revealed a *p*-value of 0.0015 with the additive model adjusted for covariates [Eq. (4)] and a *p*-value of 0.0001 with the additive model without adjusting for covariates, which was insufficient to be detected after multiple testing adjustment. We mention here that we used only the first replicate of GAW15 Problem 3 simulated data due to computational constraints, but the analysis of more replicates may have provided different results.

An important advantage of the conditional design in a real data set, is that Step 1 should provide only a small number of globally significant SNPs (i.e., two or three), which are then tested in pairwise combination with the full complement of SNPs in the study. As such, the conditional design should generate fewer interaction tests than the simultaneous design. In the GAW15 simulated data set, however, over 400 SNPs remained significant after adjustment for multiple testing in Stage 1 and caused a special situation in which the conditional design exploded into many interaction tests and lead to the physical proximity interaction effects that we detected with both approaches. It is not clear why there were so many significant results on chromosome 11 and it would be interesting to validate those results by testing the other replicates. The use of logistic regression for the detection of interactions has the disadvantage of performing poorly with high dimensionality, which can lead to false positives and decreased power. In the present study for instance, the conditional approach with additive and dominance effects required the estimation of 16,279,364 parameters from 32,154,500 observations. Another potential problem with logistic regression is the phenomenon of the separation or quasi-separation of data. This occurs in the fitting process of the logistic model when the likelihood converges while at least one parameter estimate diverges to infinity. Quasi-complete separation of data, however, was only observed in 0.3% of the tests that we performed with the additive model with covariates in this study. High correlations between predictors, referred to as multicollinearity, may also decrease the power of the logistic regression. This may occur with SNPs that are in high LD. Multicollinearity due to linkage disequilibrium could, however, be addressed statistically by combining SNPs that are in high LD into haplotypes.

## Conclusion

The significant interactions found by the full logistic regression model with both dominance and additive effects [Eq. (3)] were all captured by the additive effect coefficient (*i*_*aa*_). We conclude from our results that using a model with only the additive genetic effects of two SNPs should capture most of the underlying interactions and will reduce the multiple testing otherwise imposed by the four interaction terms. A large portion of the interactions that we detected resulted from SNP pairs that were within 1000 kb of each other. Our results clearly highlight the need to include an additional step or procedure to account for locus proximity in the evaluation of interaction effects. All remaining interactions without exception were false positives that included a SNP with exceptionally high marginal signals from chromosome 11 or chromosome 6 (Fig. 1). Those SNPs with excessively high marginal signals (*p*-values < 10^-200^) are likely to be responsible for the false-positive signals. This finding draws attention to the importance of directing further work toward the evaluation of the impact of strong marginal SNPs in interaction modeling.

## Competing interests

The author(s) declare that they have no competing interests.
